# Left-sided gall bladder: Report of two cases

**DOI:** 10.4103/0972-9941.37194

**Published:** 2007

**Authors:** R K Chrungoo, S L Kachroo, Ashwani K Sharma, Arshad Bashir Khan, Aga Syed Nadim

**Affiliations:** Department of Surgery, Govt. Medical College, Bakshi Nagar, Jammu - 180 001, (J and K), India

**Keywords:** Laparoscopic cholecystectomy, left-sided gall bladder, sinistro position

## Abstract

Left-sided gall bladder without situs inversus viscerum is a rare albeit recognized clinical entity. We report our experience of two cases of left-sided gall bladder in two women aged 36 and 48 who underwent laparoscopic cholecystectomy for chronic calculous cholecystitis. Left-sided gall bladder may provide an unusual surprise to the surgeons during laparoscopy as routine pre-operative studies may not always detect the anomaly. Awareness of the unpredictable confluence of the cystic duct into the common bile duct (CBD) and selective use of intraoperative cholangiography aid in the safe laparoscopic management of this unusual entity.

## INTRODUCTION

A left-sided gall bladder is a rare congenital anomaly defined as a gall bladder attached to the lower surface of the left lateral segment of the liver, i.e. to the left of the interlobar fissure and round ligament. This gall bladder is situated under the left lobe of the liver between segments III and IV or on segment III to the left of the falciparum ligament. Left-sided gall bladder is a para physiologic condition that, when identified before surgery, must be properly evaluated with the use of computed tomography (CT) or magnetic resonance imaging (MRI), when incidentally discovered during surgery must be promptly recognized by the surgeon who must be aware of the unpredictable confluence of the cystic duct into CBD. If in doubt, the surgeon should perform an intraoperative cholangiography. We report two adult patients in each of whom left-sided gall bladder was found incidentally, intraoperatively and managed successfully.

## CASE REPORTS

### Case 1

**Figure 1:**
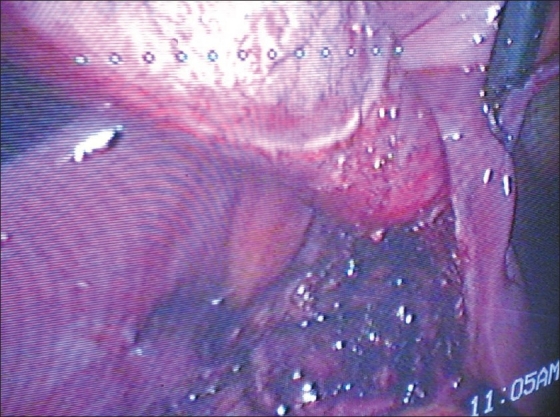
Left sided gall bladder. Retrograde dissection

A 36-year-old female presented with complaints of recurrent right upper abdominal pain associated with vomiting and flatulent dyspepsia for five months. There was no history of jaundice or hospitalization. On examination normal heart sounds were audible on left side of the chest. Abdominal examination was normal. Hemogram, kidney function tests and liver function tests were normal. Ultrasonography of the abdomen showed partially distended gall bladder with large calculus measuring 2.1 cm seen in gall bladder lumen at fundus with normal wall thickness and outline. Intrahepatic biliary radicles (IHBR) and CBD were normal. A laparoscopic cholecystectomy was planned and performed using standard 4-port technique. Pneumoperitoneum was created by closed veress needle technique. Laparoscope was introduced through a 10 mm umbilical port. Actual position of the gall bladder could not be made out because of adhesions with the omentum. 10 mm epigastric port was made and omental adhesions separated. Gall bladder was found on the left of falciform ligament [Figure 1]. Rest of the abdominal viscera were normally positioned. Two 5 mm subcostal pots were made. Callots triangle was identified. Cystic artery was found to be crossing in front of the CBD. Cystic duct opened into the common hepatic duct on its right side. The cystic artery was isolated, clipped and divided. The cystic duct was clipped and divided. Retrograde dissection of the gall bladder was done, though with difficulty. The gall bladder was extracted through the epigastric port. Postoperative course of the patient was uneventful. Histopathological examination revealed features suggestive of chronic cholecystitis.

### Case 2

**Figure 2:**
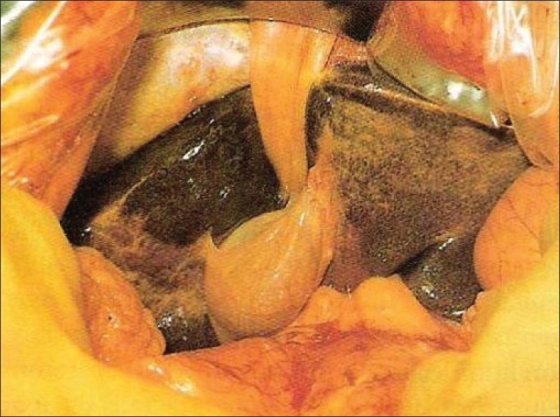
Gall bladder on left side of the falciparum ligament

**Figure 3:**
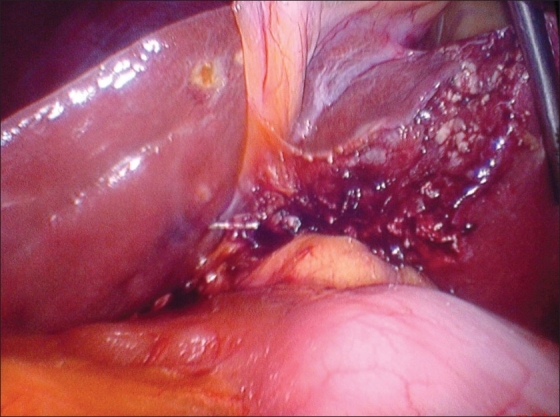
Left sided gall bladder (Removed)

A 48-year-old female was admitted in the hospital with complaint of pain in the right upper abdomen for three to four months with nausea and vomiting. There was no history of fever, jaundice and hospitalization. Liver function tests were normal. Ultrasonography showed cholelithiasis with gall bladder wall thickening. A laparoscopic cholecystectomy was performed using standard 4-port technique. The gall bladder was lying to the left of the falciform ligament [Figure 2]. The cardiac pulsation was on the left side and rest of the abdominal viscera were normally positioned. Cystic artery was found to be crossing in front of the CBD. Cystic duct opened into the common hepatic duct on its right side. The cystic artery and cystic duct were clipped and divided and retrograde dissection of gall bladder done [Figure 3]. The gall bladder was extracted through the epigastric port. The patient was discharged from the hospital on the first postoperative day. Histopathological examination revealed features suggestive of chronic cholecystitis.

## DISCUSSION

Malposition of the gall bladder occurring in the absence of situs inversus is very rare anomaly. Two types of malpositions are known (1) medioposition (2) sinistroposition (true left-sided gall bladder).[1]

In medioposition, the gall bladder is displaced medially to lie on the undersurface of the quadrate lobe (segment IV) but is still on the right side of the round ligament. In sinistroposition, the gall bladder lies under the left lobe (segment III) to the left of the round ligament. Hochstetter first described a case of sinistroposition in 1886 and since then 105 cases have been reported till 1997.[2] In a multi-center series of laparoscopic cholecystectomies, the prevalence of sinistroposition was 0.3%.[1] There are two explanations of left-sided gall bladder development: 1) The gall bladder migrates to a position under the left liver, that is, to the left of the round ligament and the location of the cystic duct is normal. 2) A second gall bladder develops directly from the left hepatic duct, accompanied by failure of development of the normal structure on the right side.

In sinistro position, the cystic artery always crosses in front of the CBD from right to the left. The cystic duct may open on the left or right side of the common hepatic duct or on to the left hepatic duct directly.[1]

As routine pre-operative studies may not detect the anomaly, it may provide the surgeons with an unusual surprise during laparoscopy. When incidentally discovered during surgery it must be promptly recognized by the surgeon, who must be aware of the unpredictable confluence of the cystic duct into CBD and should limit the use of diathermy and avoid division of structures until a clear picture of the bile duct and blood vessels is obtained.

In both our cases the condition was promptly recognized, cystic artery and cystic duct were isolated, clipped and divided and retrograde dissection of the gall bladder done. Post-operative course of the patients was uneventful in both the cases.

In selective cases adopting a French position, modification of the port sites (use of accessory ports) and the use of falciparum ligament lift may facilitate the procedure. If in doubt, the surgeon should perform an intraoperative cholangiogram to further define the biliary system. Open surgery should be considered if the anatomy still remains unclear.

## CONCLUSION

Left-sided gall bladder without situs inversus is a rare clinical entity and can provide an unusual surprise to the surgeons during laparoscopy. Awareness of unpredictable confluence of cystic duct into the CBD, selective use of intraoperative cholangiography and modification of the technique aid in safe laparoscopic management of this unusual anomaly.
